# Vector spatial and spatiotemporal laser solitons

**DOI:** 10.1515/nanoph-2024-0582

**Published:** 2025-01-20

**Authors:** Sergey V. Fedorov, Nikolay A. Veretenov, Nikolay N. Rosanov

**Affiliations:** Ioffe Institute, St. Petersburg, Russia

**Keywords:** dissipative optical solitons, laser solitons, phase and polarization singularities

## Abstract

Dissipative optical solitons, i.e. packets of radiation localized not due to the presence of optical inhomogeneities of the scheme or medium, but due to the balance of energy inflow and outflow in a nonlinear medium, deserve special attention for a number of reasons. First, these solitons are “calibrated” with a discrete set of basic parameters. This will lead to their increased stability: dissipative solitons are attractors, they are not sensitive to small perturbations. Second, progress in laser technology and the emergence of new laser and nonlinear optical materials provides an opportunity not only to study the rich physics of dissipative solitons, but also to propose their promising applications. This paper, which combines both a review of the current level of theory and original results, is devoted mainly to new types of these solitons. These types exploit the topological features of structured radiation, characteristic of vector, polarization dissipative solitons, which have a nontrivial internal structure. We sequentially present one-dimensional (1D), two-dimensional (2D) and three-dimensional (3D) polarization solitons, identify limitations in the topological protection of the information that can be encoded by topological charges and indices and discuss development prospects in this area.

## 1 Introduction

Optical solitons, which we will understand as localized due to the balance of linear spreading and nonlinear focusing in the medium of the optical radiation structure, are notable both for their rich physics and for the important applications that have already been demonstrated [1]. Optical solitons are divided into two large classes: conservative solitons, realized in transparent nonlinear media without absorption and amplification, and dissipative solitons, localization in which is caused by the balance of energy inflow and outflow. Here we will be interested only in the second class, which is also the subject of extensive literature [2], [3], [4], [5], [6], [7], [8], [9], [10], [11], [12], [13], [14], [15], [16], [17], [18], [19], [20], [21]. Dissipative optical solitons have increased stability, being, in contrast to conservative solitons, attractors.

An important type of such solitons is solitons in schemes with laser amplification and nonlinear absorption of the medium, hereinafter called laser solitons. Spatial solitons in such lasers were found in Ref. [22] and were subsequently intensively studied theoretically and experimentally. For them the theory predicts a comparative ease of obtaining not only one-dimensional, but also two- and three-dimensional solitons [16], [17], [18], [19], [20], [21]. The experimental realization of laser solitons is simpler for efficiently discrete systems, such as coupled lasers, see, e.g., [23].

Dissipative optical solitons have a nontrivial internal structure, which allows us to consider them not simply as units of information in its optical recording, but as symbols-hieroglyphs or letters of a rich alphabet. In this regard, the intersection of two modern directions is interesting: the physics of optical solitons and singular or topological optics [24], [25], [26]. The latter direction has been most thoroughly studied for scalar solitons, that is, radiation structures with a fixed and unchanging polarization. The objective of this article is to analyze vector, polarization laser solitons and polarization singularities in them. Naturally, the involvement of the polarization degree of freedom expands the arsenal of optical solitons and presents new possibilities in recording, transmitting and processing information. Singularities, being topological features, deserve special attention due to the topological protection of the information recorded on their basis.

In this paper, we restrict ourselves to the theory of vector laser solitons; a review of the experimental methods and applications can be found in Ref. [27]. Next, in Section 2, we present the models under consideration, introduce the general governing quasi-optical (paraxial) equation for the envelopes, and discuss its properties. Sections 3–5 are devoted, successively, to 1D, 2D, and 3D vector polarization solitons. The main conclusions are given in Section 6.

## 2 Models and governing equations

We consider a model of a linear matrix with embedded centers that have two resonant transitions. In semiconductors, the role of levels between which these transitions occur can be played by zones. The radiation is considered as a classical field, with quantum electrodynamic effects neglected. Within the framework of the quasi-optical approximation for slowly changing envelopes of waves with opposite circular polarizations *E*_±_, the governing equation has the following dimensionless form:

(1) $$\frac{\partial E_{\pm }}{\partial \zeta }=\overset{D}{\underset{j=1}{\sum }}\left(i+d_{j}\right)\frac{\partial^{2}E_{\pm }}{\partial x_{j}^{2}}+f_{\pm }E_{\pm }.$$

Here *D* = 1, 2, 3 is the dimension of the scheme, *ζ* = *t* or *z* or *τ* = *t* − *z*/*v*_*g*_, *x*_1_ = *x*, *x*_2_ = *y*, *x*_3_ = *z* or *x*_3_ = *τ*, *x*, *y*, *z* are the Cartesian coordinates, *t* is the time, *v*_*g*_ is the group velocity in the matrix without active centers, *f*_±_ is the optical response of the active centers and the matrix. The evolutionary variable *ζ* normalization is performed using characteristic length or time associated with non-resonant linear losses, and the normalization of other quantities is different for different schemes and is indicated in the original works. The envelopes of the Cartesian and circular polarization components are related to the envelopes for the Cartesian polarization components by the relations $E_{\pm }=\left(E_{x}\pm iE_{y}\right)/\sqrt{2}$ .

**Figure 1:**
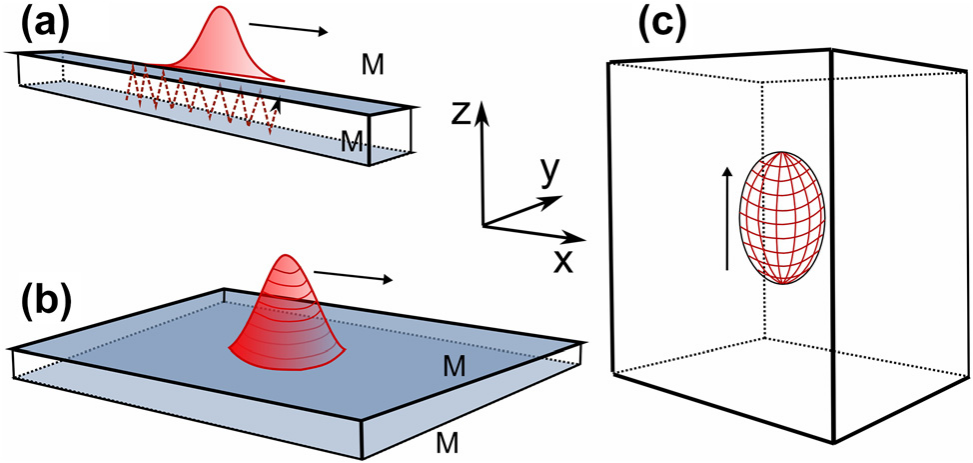
Schemes in which one-dimensional (a), two-dimensional (b) and three-dimensional (c) laser solitons are formed. The media consist of a matrix with embedded active (laser gain) and passive (saturable absorption) centers. M are mirrors. Localized structures with straight arrows are the soliton intensity distributions (red). In (a), the dotted line shows the ray path.

The dimensionality of the scheme *D* is determined by the number of spatial coordinates along which the structure is limited by nonlinearity rather than by external inhomogeneity. It is illustrated in Figure 1. In all variants, the radiation propagates predominantly along the axis *z*. The one-dimensional variant *D* = 1 (Figure 1(a)) as applied to spatial structures corresponds to a wide-aperture slit interferometer, inside which the above-mentioned medium is located. In this case *ζ* = *t*. At *D* = 2 (Figure 1(b)), the interferometer is two-dimensional and still *ζ* = *t*. 3D-structures limited in all coordinates, strictly speaking, can be obtained in a resonator-free scheme of great length (Figure 1(c)), and then the evolutionary variable *ζ* = *z*. Finally, all these schemes described by equation (1) can be placed in a ring resonator of great length, so that the time it takes for light to travel through it exceeds the characteristic durations of the structure and the relaxation times.

“Diffusion coefficients” *d*_*j*_ reflect the angular selectivity of losses, $d_{⊥}=\left(d_{1},d_{2}\right)$ , and frequency selectivity, *d*_3_ = *d*_*τ*_. In the main text we will assume that *d*_1_ = *d*_2_ = *d*_3_. The quantities *f*_±_ reflect the optical properties of centers with laser amplification and with saturable absorption. They also include non-resonant absorption in the matrix:

(2) $$f_{\pm }=-1-\left(1-i\delta_{a}\right)a+\left(1-i\delta_{g}\right)g_{\pm }.$$

The term −1 on the right-hand side of relation (2) represents normalized non-resonant losses, *δ*_*a*,*g*_ are frequency detunings of the centers of the absorber and amplifier spectra relative to the carrier frequency (in semiconductor lasers this is the so-called *α*-factor), *a* and *g* are complex absorption and gain. When relaxation processes are taken into account, they are determined by the Bloch equations. For a saturable absorber, it is sufficient to use a two-level model of the medium [22]. In the absence of frequency detunings, the dynamics of material absorption obeys the equation

(3) $$\tau_{a}\frac{\partial a}{\partial t}=-\left(1+b_{a}I\right)\left(a-a_{s}\right),\;a_{s}=\frac{a_{0}}{1+b_{a}I}.$$

Here *τ*_*a*_ is the relaxation time of resonant absorption, *I* = *I*_+_ + *I*_−_ is the total intensity of right $I_{+}={\left|E_{+}\right|}^{2}$ and left $I_{-}={\left|E_{-}\right|}^{2}$ circular polarizations, *b*_*a*_ is the inverse saturation intensity of absorption, *a*_0_ is the absorption in the limit of weak intensity, *a*_*s*_ is the steady-state value of resonant absorption in the case of monochromatic radiation with intensity *I*.

To describe the gain in vertical cavity semiconductor lasers, a four-level model of a medium with two working transitions is adopted with the values of the longitudinal component of the total angular momentum for the first transition *J* = 1/2 and 3/2 and for the second *J* = −1/2 and −3/2 (spin-flip model). Denoting by *n*_*J*_ the quantity proportional to the population of the level with longitudinal angular momentum *J*, we introduce combinations of populations $N=\left[\left(n_{1/2}+n_{-1/2}\right)-\left(n_{3/2}+n_{-3/2}\right)\right]/2$ and $n=\left[\left(n_{-1/2}+n_{-3/2}\right)-\left(n_{1/2}+n_{3/2}\right)\right]/2$ , as well as the difference in the intensities of the circular components *δI* = *I*_−_ − *I*_+_. Then *g*_±_ = *N* ± *n*, and the gain dynamics is determined by the equations

(4) $$\begin{array}{c} \tau_{g}\frac{\partial N}{\partial t}=-\left(1+b_{g}I\right)\left(N-N_{s}\right)+b_{g}\delta I\left(n-n_{s}\right),\\ \varepsilon_{J}\tau_{g}\frac{\partial n}{\partial t}=-\left(1+\varepsilon_{J}b_{g}I\right)\left(n-n_{s}\right)+\varepsilon_{J}b_{g}\delta I\left(N-N_{s}\right). \end{array}$$

In (4) the quantities *τ*_*g*_ and *b*_*g*_ are analogous to those in the passive medium for the amplifying medium, *ɛ*_*J*_ is the ratio of the relaxation times of the four-level scheme, and for the indicated values this parameter is positive and small [28]. The steady-state values *N* and *n* have the form

(5) $$\begin{array}{c} N_{s}\left(I,\delta I\right)=g_{0}/\left(1+b_{g}I-\frac{\varepsilon_{J}b_{g}^{2}\delta I^{2}}{1+\varepsilon_{J}b_{g}I}\right),\\ n_{s}\left(I,\delta I\right)=\frac{\varepsilon_{J}b_{g}\delta I}{1+\varepsilon_{J}b_{g}I}N_{s}\left(I,\delta I\right). \end{array}$$

When *ɛ*_*J*_ = 0, it follows from (4) and (5) that *n* = 0 and for *N* we arrive at an effectively two-level model of type (3). Note also that the parameter *ɛ*_*J*_ is included in (5) as a factor of the difference in intensities *δI*. Therefore, for modes with linear polarization, for which *ɛ*_*J*_ = 0, the dependence of nonlinearity disappears and the model is again reduced to a two-level one.

According to (1), (3) and (4), the nonlinear relationship of the polarization components is incoherent, i.e. not sensitive to the phases of the envelopes for these components. The governing equations are invariant to the replacement *E*_+_ ↔ *E*_−_, which reflects the equality of circular polarizations in an isotropic medium. In the special case of identical diffusion coefficients *d*_*j*_ = *d*, the form of the sum in (1) is replaced by $\left(i+d\right)L_{D}$ , where *L*_*D*_ is the *D*-dimensional Laplacian; this leads to additional symmetry.

Localized structures are conveniently characterized by the following quantities: energy

(6) $$W_{D}\left(\zeta \right)=\underset{D}{\int }I\left(x,\zeta \right)\text{d}x,$$

energy flows (Poynting vector)

(7) $$S\left(x,\zeta \right)=I_{+}\nabla \Phi_{+}+I_{-}\nabla \Phi_{-},\;\Phi_{\pm }=\arg E_{\pm },$$

vector of coordinates of the radiation power or energy center

(8) $$R\left(\zeta \right)=\underset{D}{\int }xI\left(x,\zeta \right)\text{d}x/W_{D}\left(\zeta \right),$$

moment of inertia tensor

(9) $$J_{kl}\left(\zeta \right)=\underset{D}{\int }\left(x_{k}^{2}\delta_{kl}-x_{k}x_{l}\right)I\left(x,\varsigma \right)\text{d}x.$$

The definition (9) is appropriate for 2D and 3D solitons. Stabilization of these values when calculating the evolution of the structure serves as a sign of the establishment of a rigid soliton.

The polarization characteristics of the structures are specified by the Stokes parameters

(10) s0=I,s1=2ReE−∗E+,s2=−2ImE−∗E+,s3=δI.

Topological singularities in the considered schemes can be of two types. Firstly, these are wave front singularities at *s*_0_ = 0 due to the uncertainty of the phase for zero intensity. Secondly, these are polarization singularities: V-points at the uncertainty of polarization in the case of *s*_0_ = 0, L-points at purely linear polarization (*s*_3_ = 0, the direction of rotation of the electric field vector along the polarization ellipse is not defined), and C-points (uncertainty of the direction of the main axis of the polarization ellipse at purely circular polarization, *s*_1_ = *s*_2_ = 0). On the Poincaré polarization sphere, the C-points are depicted as poles, and the L-points lie on the equator. The set of singularity points can form lines, surfaces and even fill volumetric regions depending on the dimensionality of the problem. Polarization singularities are characterized by the Poincaré index *η*, defined as the number of revolutions of the polarization ellipse when going around the singular point along a closed contour of small dimensions.

For class A lasers (limit of small relaxation times), such lasers, in particular, in the cases under consideration can include quantum cascade lasers [29], [30] the characteristics of the medium instantly follow the field. In this case, in (2) instead of *a* and *g* one should understand their values established under the current radiation characteristics:

(11) $$f_{\pm }=-1-\left(1-i\delta_{a}\right)a_{s}+\left(1-i\delta_{g}\right)\left(N_{s}\pm n_{s}\right).$$

Here we are interested only in bright laser solitons, on the periphery of which the envelopes vanish. Therefore, the non-generation mode must be stable, which is achieved when

(12) $$-1-a_{0}+g_{0}<0.$$

Thus, the excitation of bright dissipative solitons can only be hard, insufficiently strong perturbations vanish during evolution.

The results shown in the figures below were obtained by numerical calculation with fixed parameters *a*_0_ = 2, *b*_*g*_ = 0.1, and the intensity was normalized to the saturation intensity, *b*_*a*_ = 1. The initial condition was based on the use of the corresponding scalar solitons.

## 3 1D schemes

For the one-dimensional resonator scheme shown in Figure 1(a), the evolutionary variable is time *t*. In the inertialess case, the control equation (1) takes the form

(13) $$\frac{\partial E_{\pm }}{\partial t}=\left(i+d\right)\frac{\partial^{2}E_{\pm }}{\partial x^{2}}+\left(-1-a_{s}+N_{s}\pm n_{s}\right)E_{\pm }.$$

**Figure 2:**
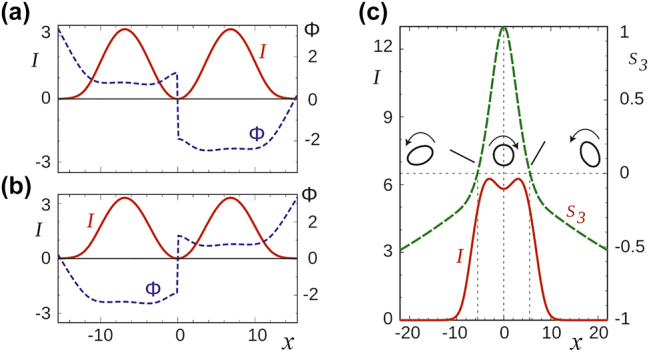
The intensity (red, solid lines) and phase (blue, dotted) profiles of the circular *E*_+_ and *E*_−_ components are shown in (a) and (b), respectively. In (c), the profile of the total intensity (red line) and the Stokes parameter S_3_ (green dotted line) of the combined vector soliton are shown. The ellipses show the instantaneous polarization state at three points of the soliton. The ellipses rotate with time due to the difference in frequencies of the parent scalar solitons and correspondingly soliton polarization components.

We will choose as circular polarization components known scalar solitons, which can be of two types [31]: fundamental, with a bell-shaped intensity distribution, the envelope of which, symmetrical relative to the center, does not vanish at finite distances from the center, and a soliton with a single zero of intensity in the center of the structure (Figure 2, panels (a) and (b) correspond to two circular polarization components of a vector soliton). If one chooses the same scalar soliton for both components, then *δI* = 0 for it, therefore *n*_*s*_ = 0. Accordingly, localized structures of this type satisfy equation (13).

Their stability is confirmed both by the direct numerical solution of equation (13) when choosing scalar solitons or structures close to them with the addition of weak noise for the field components as the initial condition, and by the linear stability analysis. However, such solitons have linear polarization everywhere, L-points fill the entire space; an exception is the variant of scalar solitons with zero intensity at the center, which serves as a V-point for a vector soliton. Another reservation concerns the degenerate case *ɛ*_*J*_ = 0, in which there are solitons with an arbitrary ratio of component intensities with their fixed sum (indifferent equilibrium). With a difference in intensities, the polarization is elliptical and the same in the entire space. But in this case, localized structures are not attractors, the soliton parameters drift under the action of noise, so it is difficult to speak of their stability. With the slightest difference *ɛ*_*J*_ from zero, the component intensities equalize over time. Such a soliton can already be considered stable (an attractor). Therefore, further in this version we will understand *ɛ*_*J*_ in the ultimate sense, *ɛ*_*J*_ → +0.

Less trivial is the polarization structure of a combined vector soliton, one of whose components is close to the fundamental soliton, and the other to the soliton with zero intensity at the center (Figure 2(c)), and the centers of the components coincide. An established vector soliton is “rigid”, i.e. the field distribution is not deformed with time and only their shifts as a whole are allowed. In this case, there is inversion symmetry and, in accordance with the rules of Euler mechanics [32], the soliton is motionless (there is no its motion along *x*). The polarization of the radiation is non-uniform over *x* and elliptical, with three singularity points: a С-point in the center (*x* = 0) and two L-points located symmetrically.

As noted above, the dynamics of temporal solitons in active microresonators can be also described by an equation of the form (13), with a natural change of variables. Therefore, many of the results presented in this section are also applicable to them.

## 4 2D schemes

In the 2D resonator scheme, the longitudinal coordinate *z* also serves as the evolutionary variable. Assuming that the medium is isotropic, i.e. its properties do not depend on the direction in the plane (*x*, *y*), we write the governing equation (1) for such schemes in the form

(14) $$\frac{\partial E_{\pm }}{\partial t}=\left(i+d\right)\nabla_{⊥}^{2}E_{\pm }+f_{\pm }E_{\pm },\;\nabla_{⊥}^{2}=\frac{\partial^{2}E_{\pm }}{\partial x^{2}}+\frac{\partial^{2}E_{\pm }}{\partial y^{2}}.$$

In the inertialess version, (12) must be taken for *f*_±_, and when relaxation is taken into account, the Bloch equations (3) and (4) must be used.

Bright scalar 2D-solitons with different topological charges *m* are known [19], [32], [33]; in the general case, the integer topological charge of an isolated singular point is defined as the phase shift when going around the point, divided by 2*π*. Their envelope in polar coordinates (*r*, *φ*) has the form

(15) $$E_{m}\left(r,\varphi ,t\right)=A_{\left|m\right|}\left(r\right)\exp\left(im\varphi -i\omega_{\left|m\right|}t\right).$$

The asymptotics of the amplitude for small *r* is as follows: *A*_|*m*|_(*r*) ∼ *r* ^|*m*|^. At the periphery the amplitude decreases approximately exponentially. The nonlinear frequency shift *ω*_|*m*|_, like the amplitude *A*_|*m*|_(*r*), depends only on the modulus of the topological charge. The stability regions of such solitons with an axisymmetric intensity distribution partially overlap, narrowing with increasing of $\left|m\right|$ .

If we choose such solitons as circular polarization components of the vector structure, then in the degenerate case *ɛ*_*J*_ = 0, as for 1D schemes, there is a family of vector solitons with the component profile (15) and an arbitrary ratio of their intensities with a total intensity coinciding with the intensity of the generating scalar soliton. With a difference in intensities, the polarization is elliptical and the same in the entire space. As already indicated, these structures are not attractors, passing from one to another under the action of weak noise.

### 4.1 Inertialess medium

One-component (*E*_+_ ≡ 0 or *E*_−_ ≡ 0), whose non-zero component is a scalar soliton, turn out to be unstable [34]. In general, in numerical calculations, scalar solitons can serve as initial distributions of the envelopes of circular polarization components.

**Figure 3:**
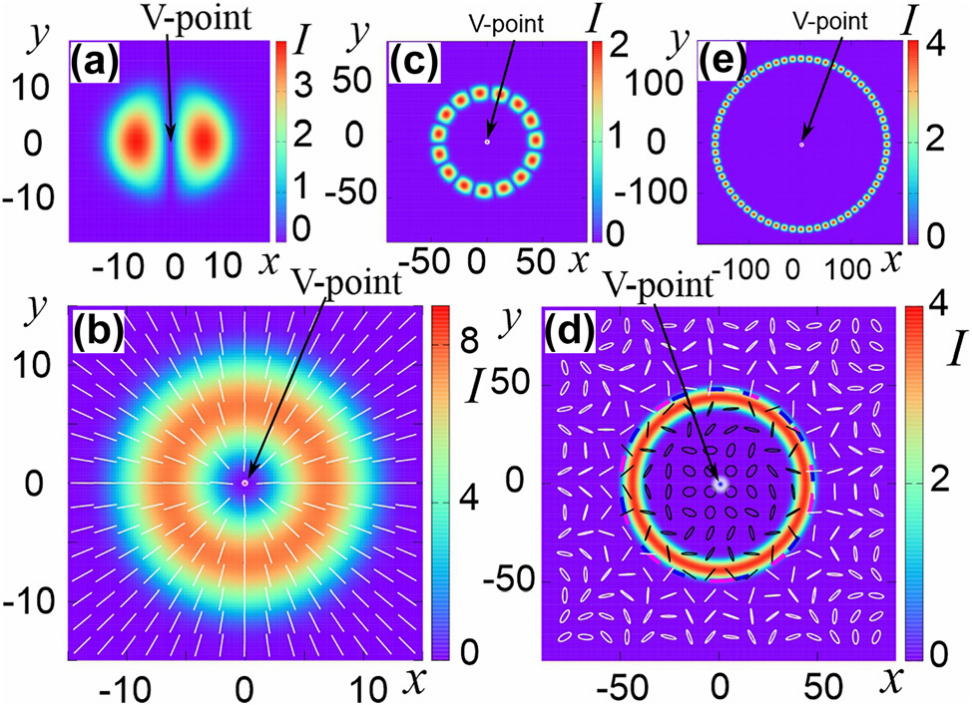
Intensity distribution for the Cartesian component I_x_ (a, c, e) and the polarization structure of the field (b, d) of vector solitons with topological charges of the circular components (1, −1) (a), (−3, 12) (b, c, d) and (32, −32) (e).

If the centers of the generating scalar solitons coincide, then their symmetry is preserved in the established vector soliton. Examples of such symmetric vector solitons are shown in Figure 3.

Stable vector solitons with different topological charges of the components constitute a large family. As shown by the linear stability analysis and confirmed by the numerical calculation, the stability of solitons with the same signs of the moduli of the topological charges of the polarization components, $\left|m_{+}\right|=\left|m_{-}\right|$ , is determined by two factors. The first is the coincidence or opposition of the directions of the azimuthal energy flows. The second is the value of the coupling parameter of the resonant levels of the laser medium *ɛ*_*J*_. The second factor, an increase in *ɛ*_*J*_, contributes to an increase in stability. The first factor shows a tendency to an increase in the stability of solitons with opposite directions of the azimuthal energy flows, that is, at *m*_+_ = −*m*_−_.

For a fundamental soliton, *m*_+_ = *m*_−_ = 0, only the second factor acts, therefore such a soliton is stable, as are solitons with opposite signs of charges, *m*_+_ = −*m*_−_. For symmetric solitons with charges of polarization components $\left|m_{+}\right|=\left|m_{-}\right|=m$ the width of stability region in the parameter *g*_0_ (linear gain or pumping) narrows, with shifting the stability region from that for a scalar 2D soliton to 1D soliton, with increase of $\left|m\right|$ . This is due to the fact that the intensity localization region of such solitons is a ring with a radius increasing with $\left|m\right|$ . Thus, the ring becomes locally close to the strip and the intensity profile of the polarization components approaches the profile for a 1D soliton for large $\left|m\right|$ . For coinciding nonzero charges, *m*_+_ = *m*_−_ ≠ 0, there is a stability threshold for *ɛ*_*J*_. At subthreshold values of *ɛ*_*J*_, the second factor is weaker than the first, and such solitons are unstable. Above the threshold of *ɛ*_*J*_, the effect of the second factor prevails, and such solitons are stable.

In Ref. [34], [35], stable solitons with coinciding and differing component centers and topological charge moduli up to a topological charge of 32 were numerically found, see Figure 3(e). The Poincaré index of symmetric structures

(16) $$\eta =\left(m_{+}-m_{-}\right)/2.$$

**Figure 4:**
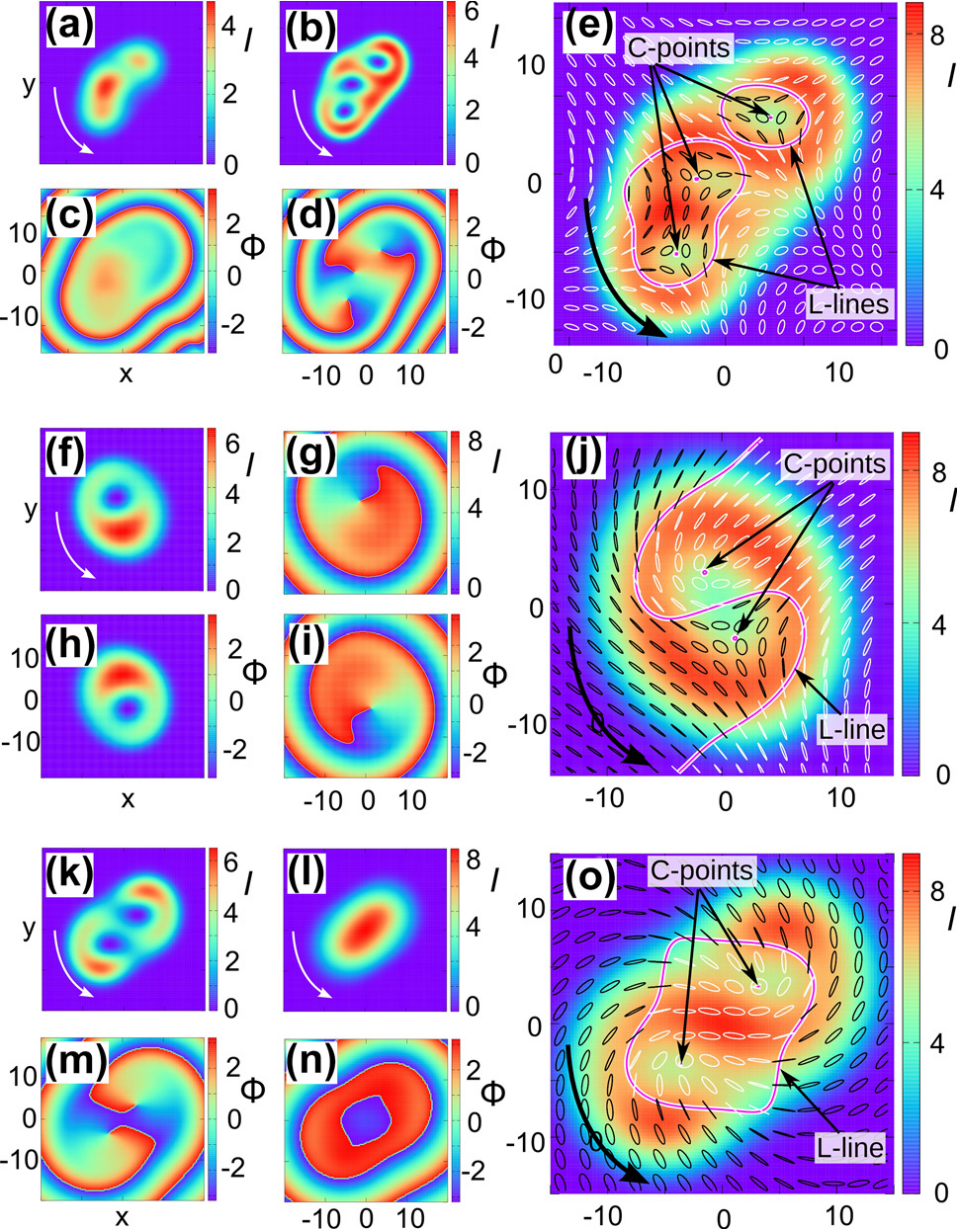
Instantaneous profiles of intensity *I* and phase Φ of circular components of the soliton with topological charges (0, 3) with Poincaré index *η* = −3/2, as well as the polarization structure (e); the same for the soliton with charges (1, 1) with non-coinciding centers and *η* = 0 (f–j); the same for the soliton with charges (2, 0) and *η* = 1 (k–o).

When the moduli of the charges of the generating scalar solitons differ, their frequencies also differ, so the established polarization structure rotates with time with a period corresponding to the difference in these frequencies. They are illustrated both in Figure 3 above for coinciding centers of the components, and in Figure 4 for different centers. In the latter case, the vector soliton is sharply asymmetric. Nevertheless, the field distribution of the soliton illustrated in Figure 4(f–j) with two isolated C-points and without V-points has inverse symmetry. Therefore, in accordance with the rules of “Euler mechanics”, the soliton rotates with a constant angular velocity around the center of symmetry. Finally, solitons with polarization component charges 0 and *m*, where $\left|m\right|=1,2$ and 3 are stable at *ɛ*_*J*_ > 0.02. At *ɛ*_*J*_ = 0, the corresponding initial structures are destroyed with the establishment of a nonlasing regime everywhere.

The variant of the structure established when scalar solitons are generated by charges and non-coinciding centers *m*_±_ = 1 (Figure 4(f–o)) deserves attention. In this case, the transverse field distributions of the polarization components are also asymmetric, but the vector soliton rotates around a fixed center. This is caused by a new symmetry, specific to vector structures, of spatial inversion with a simultaneous permutation of components *E*_+_ ↔ *E*_−_. An partial set of found solitons is shown in Table 1.

**Table 1:** Polarization characteristics of the found 2D-solitons for the corresponding values of *ɛ*_*J*_.

*m*_+_	*m*_−_	Poincare index	Polarisation structure
0	1	0.5	C-point, L-circle
0	2	1	2 C-points, L-curve
0	3	1.5	3 C-points, 2 L-curves
1	1	0	2 C-points, L-curve
1	−1	1	V-point, linearly polarized
2	−2	2	V-point, linearly polarized
3	−2	2.5	V-point, L-circle
3	−3	3	V-point, L-circle
12	−3	7.5	V-point, L-circle
12	6	3	V-point, L-circle
32	−32	32	V-point, linearly polarized

### 4.2 Effect of relaxation and other additional factors

“Standard” semiconductor lasers belong to class B, their dynamics are significantly affected by relaxation processes. Relaxation does not affect the characteristics of stationary modes, described by solutions of equation (14) with $\frac{\partial E_{\pm }}{\partial t}=0$ , but changes the boundaries of the parameter regions corresponding to their stability.

**Figure 5:**
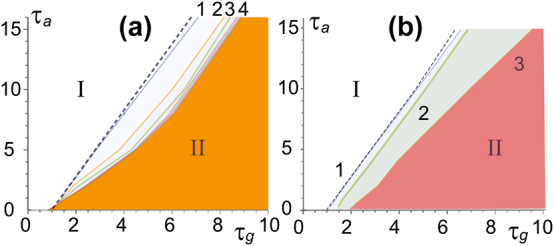
Stability domains (I) of solitons with radially symmetric intensity distribution on the lane of relaxation times for amplification *τ*_*g*_ and resonant absorption *τ*_*a*_. The dash curve is the boundary of stability for the scalar soliton with charge *m* = 1. Curve 1 is the stability boundary for the vector soliton with unit Poincaré index and components’ charges *m*_+_ = 1, *m*_−_ = −1. (a) Boundaries for zero detuning, *δ*_*g*_ = *δ*_*a*_ = 0, and *η* = *m*_+_ = 2, *m*_−_ = −2 (curve 2), *η* = *m*_+_ = 3, *m*_−_ = −3 (curve 3); curve 4 is the boundary for graphically coinciding regions for *η* = *m*_+_ = −*m*_−_ = 6, 8, 12, …, ∞. (b) Boundaries for a soliton with for frequency detunings *δ*_*g*_ = *δ*_*a*_ = 0, 2 (curve 2) and *δ*_*g*_ = 0.2, *δ*_*a*_ = 0.

As was indicated in Section 2, for vertical-cavity lasers we use the spin-flip model and equation (4). The results of the stability analysis for solitons with topological charges of the polarization components *m*_+_ = *m* = −*m*_−_ are as follows (Figure 5(a)) [34], [38]. In the plane of parameters – the relaxation times of gain and absorption – the stability region of a vector soliton with *m* = 1 is wider than that of a scalar soliton with the same topological charge. This is caused by mutual support of polarization components with oppositely directed azimuthal energy flows. At *m* ≫ 1 the stability region approaches that for 1D scalar solitons. Indeed, in this case the maximum field intensity is concentrated in a ring whose radius increases with growth of *m*. Therefore, the ring turns out to be locally close to a 1D strip.

Similarly, the effect of frequency detunings (relation (2)) is seen from Figure 5(b). The figure, in comparison with Figure 5(a), shows that the stability region of a vector soliton with component charges *m*_+_ = 1 = −*m*_−_ increases with increasing detunings. Note also that the shift of the stability boundary can be compensated by a simultaneous increase in detunings *δ*_*a*_ and *δ*_*g*_.

Anisotropy of the medium, i.e. the difference in its optical properties depending on the direction in the plane (*x*, *y*), can be caused by mechanical stresses [36]. With regard to vector solitons, the most important is the difference in the principal values of the refractive index (birefringence). This factor leads, on the one hand, to an increase in the coupling of the polarization components and, on the other hand, to a weakening of the coupling due to an increase in their frequency splitting. Numerical calculations [37] show that at subcritical values of birefringence, the frequency locking regime is realized with linear inhomogeneous field polarization.

**Figure 6:**
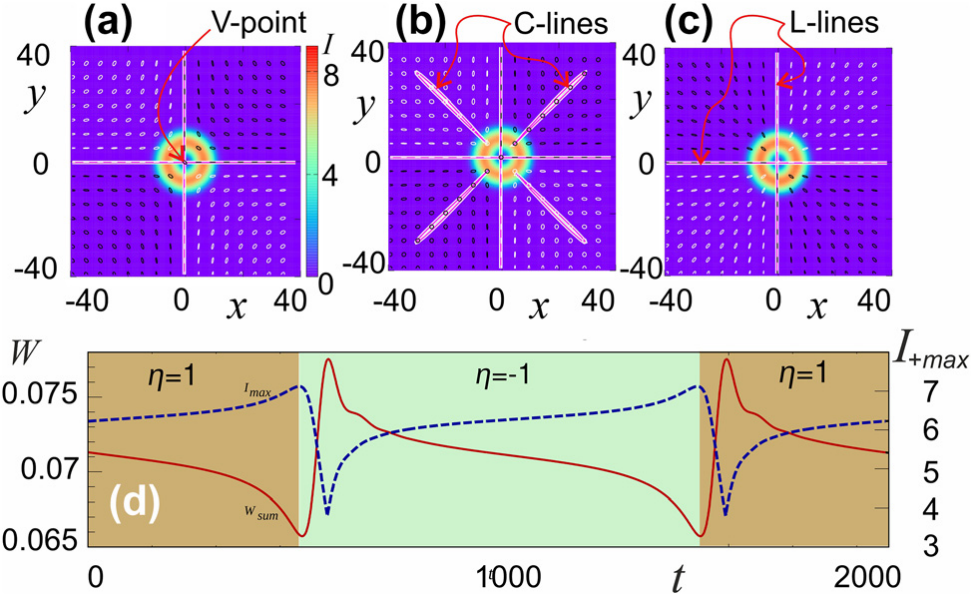
Effect of anisotropy for 2D solitons. (a–c) Variation of polarization structure of soliton with initial topological charges of the components are *m*_+_ = 1, *m*_−_ = −1 in the vicinity of their alternation. (d) Temporal variation of soliton total power *W* (solid curve, red) and maximum intensity of a circular polarization component *I*_+_ (dashed line, blue) on nearly one period of soliton oscillations.

Above the threshold, the polarization becomes elliptical. Exiting the frequency locking region with increasing birefringence leads to an interesting regime of periodic changes in the polarization structure of the soliton, including alternation of topological charges and the Poincaré index (Figure 6).

The question of realistic methods for creating such vector solitons is also important. It seems that the most accessible for the experiment is the temporary switching on of holding radiation with the corresponding charges for the polarization components [34]. In this case, the generalization of (14) is the governing equation

(17) $$\frac{\partial E_{\pm }}{\partial t}=\left(i+d\right)\nabla_{⊥}^{2}E_{\pm }+f_{\pm }E_{\pm }+E_{in,\pm }.$$

**Figure 7:**
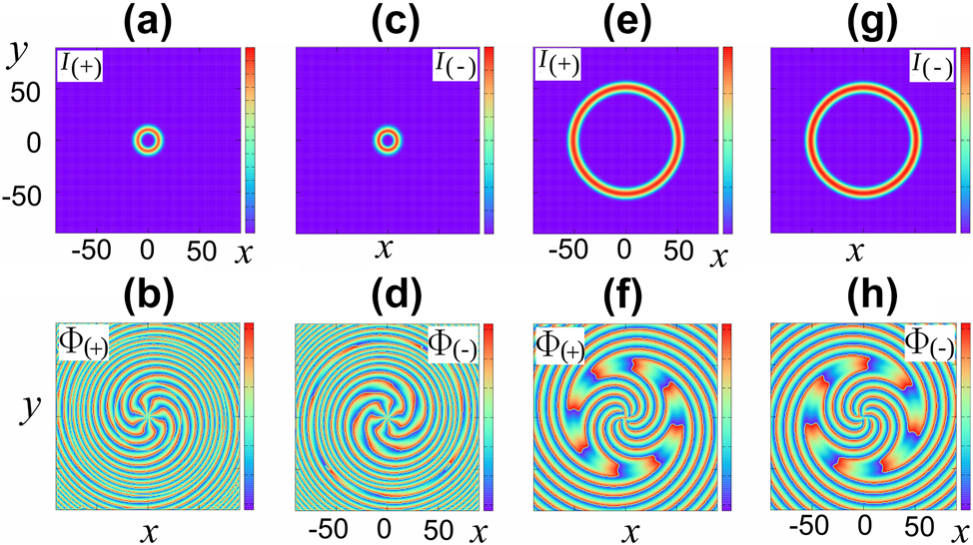
Top row: transverse intensity distributions of the circular polarization components of the soliton with the holding beam turned on (a, c) and in the steady-state mode after the beam is turned off (e, g). Bottom row: corresponding phase distributions.

The introduction of holding radiation with envelopes *E*_in,±_ allows one to control the topology of the soliton and its characteristics. The result of switching of the initial soliton to the desired one is shown in Figure 7.

## 5 3D schemes

For 3D schemes, we use governing equation (1) with dimension *D* = 3, evolution variable *ζ* = *z* and *x*_3_ = *τ*:

(18) $$\frac{\partial E_{\pm }}{\partial z}=\overset{3}{\underset{j=1}{\sum }}\left(i+d_{j}\right)\frac{\partial^{2}E_{\pm }}{\partial x_{j}^{2}}+f_{\pm }E_{\pm }.$$

As before, for these schemes, bright (with finite energy) localized structures can be stable only if the unsaturated losses exceed the unsaturated gain, i.e. *f*_±_(*I*_±_ = 0) < 0. In other words, only hard excitation of the solitons of interest to us is possible.

The general method and approximate scenario for the formation of the two types of solitons considered in this section are as follows. The generating ones are two (one for each circular polarization) localized toroidal structures with topological charges *m*_+_ and *m*_−_, regardless of their stability; moreover, under realistic conditions for laser schemes, they are unstable. More exactly, for *m*_+_ = *m*_−_ = 0 these intensity distributions are not toroidal (intensity of toroidal ones vanishes at least on a line in a non-simply connected region), but scheroidal, with non-vanishing total intensity everywhere, in a simply connected region. Such structures can be stable. However in this case the polarization is uniform in space and effectively these structures are scalar and therefore we will not consider them below. The two tori are located close to each other and their symmetry axes are located at some angle to each other. Numerical simulation shows that there is a critical value of this angle.

The critical value depends significantly on the parameter *ɛ*_*J*_. In the considered range of parameters, the critical angle is 25° for the typical value in calculations *ɛ*_*J*_ = 0.04, and it decreases to 7° for *ɛ*_*J*_ → 0.

At subcritical values of the initial angle, the tori gradually become parallel and spatially merge. This is the case of toroidal vector laser solitons. At supercritical values of initial angle, this angle gradually increases up to a value of *π*/2. In this variant, “cruciform” cross-shaped vector laser solitons are formed. The presentation is based on the papers [38], [39], [40].

Next, in Section 5.1, examples are presented and properties of toroidal solitons are discussed. The following Section 5.2 is devoted to cruciform solitons and transitions between these two types of solitons. We will start with a simpler version of inertialess nonlinearity, when functions *f*_±_ have the form (12), and the values of the “diffusion coefficients” coincide, *d*_*j*_ = *d*. In this case, the space **x** = *x*_1_, *x*_2_, *x*_3_ is isotropic according to (18). Some additional factors, including consideration of relaxation processes, are considered in Section 5.3.

### 5.1 Toroidal solitons

For toroidal solitons, the initial field distribution in numerical simulation is formed by rotating the field of a scalar 2D symmetric soliton with a proper topological charge. The center of the scalar soliton is located at some distance from the rotation axis, and for definiteness we consider the rotation axis to be the axis *x*_3_ = *τ*.

Table 2 describes the topological singularities (topological charges of the circular polarization components *m*_+_ and *m*_−_ and the lines or surfaces of the singularities and their number) of a number of found 3D vector toroidal solitons of the lowest order. The solitons presented in Table 2 are “rigid”; their shape does not change during propagation, except for displacements and rotations on the whole. Below we will comment on the structure of some of these solitons.

**Table 2:** Polarization features of toroidal solitons.

*m*_+_	*m*_-_	*ε*_*J*_	Polarization singularities and symmetry
1	0	0.04	C-line, cylindrical symmetry, L-surface
1	−1	0.0	V-line, linear polarization
2	−1	0.04	V-line, cylindrical symmetry, L-surface
3	−1	0.04	V-line, L-surface
2	−2	0.04	V-line, linear polarization
3	−2	0.04	3 C-lines, L-surfaces
3	−3	0.04	V-line, linear polarisation
4	−2	0.04	6 C-lines, L-surfaces
4	−3	0.08	7 C-lines, L-surfaces
4	−4	0.1	4 V-lines, L-surfaces
5	−5	0.1	1 V-line, 8 C-lines, L-surfaces
5	−1	0.12	1 V-line, 2 double C-lines, L-surfaces
6	−1	0.06	7 C-lines, L-surface
7	−1	0.08	8 C-lines, L-surfaces
7	−5	0.12	1 V-line, 12 C-lines, L-surfaces

#### 5.1.1 Axisymmetric toroidal solitons

**Figure 8:**
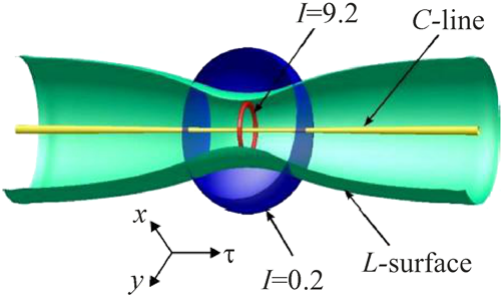
Two isointensity surface, *I* = 9.2 and 0.2, C-line and L-surface of symmetric toroidal soliton with the topological charges of components *m*_+_ = 1 and *m*_−_ = 0.

The simplest structure is that of a soliton with charges of circular polarization components *m*_+_ = 1 and *m*_−_ = 0. For this soliton, Figure 8 shows isointensity surfaces at two levels of total intensity and polarization singularities – the C-line (straight line, axis *τ*, the polarization is right circular on it) and the L-surface with axial symmetry and the C-line as the axis of symmetry. At other points the polarization is elliptical and changes in space while maintaining axial symmetry. The total intensity *I* is maximum, *I*_max_ ≈ 9.2, in the region of a wider isointensity surface. The intensity decreases with distance from this surface both outward and inward. In the latter case, it does not vanish, its local minimum *I*_min_ ≈ 0.2 is reached near the other surface shown in the figure. There are no V-points, since the total intensity vanishes only asymptotically at the periphery of the soliton.

**Figure 9:**
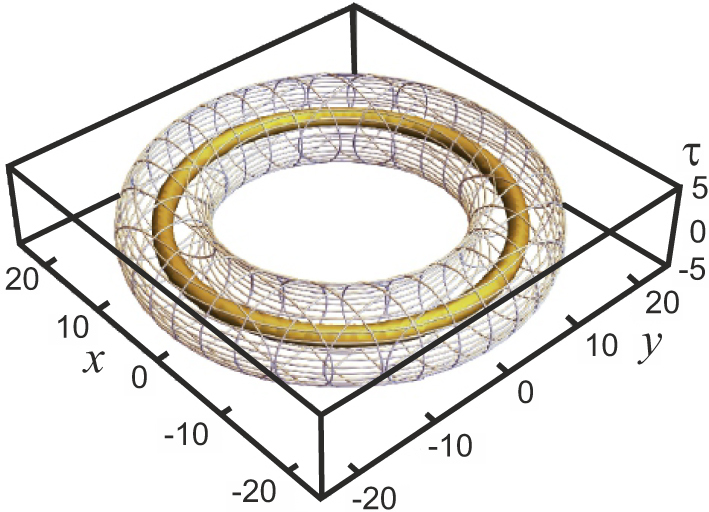
Toroidal isointensity surfaces at levels 1 (internal, mesh) and 8.5 (external, solid) of a toroidal soliton with topological charges of components *m*_+_ = 4 and *m*_−_ = −4. The field polarization is linear everywhere.

Other symmetric structures are formed at $1\leq \left|m_{+}\right|=\left|m_{-}\right|\leq 3$ . When the moduli of the topological charges of the polarization components coincide, the C-line serving as the axis of symmetry becomes degenerate with multiplicity $\left|m_{+}\right|=\left|m_{-}\right|$ . At $\left|m_{+}\right|=\left|m_{-}\right|>3$ it splits into $\left|m_{+}\right|=\left|m_{-}\right|$ non-degenerate (single) ones. In this case, strict axial symmetry is broken and, for example, at *m*_+_ = 4 and *m*_−_ = −4 it becomes an axis of symmetry of the fourth order. This soliton is shown in Figure 9.

Table 2 also lists other variants of combinations of topological component charges that form symmetric solitons. The symmetry axis is the V-line for solitons with nonzero component charges and the C-line otherwise. Solitons are rigid, i.e. their shape does not change during propagation up to displacements and rotations. The stability of solitons depends significantly on the parameter *ɛ*_*J*_. Its “standard” value in calculations is *ɛ*_*J*_ = 0.04. For smaller values, up to *ɛ*_*J*_ = 0, only a soliton with charges *m*_+_ = −*m*_−_ = 1 (and its mirror image) is stable. For solitons with higher component charges, the critical value of *ɛ*_*J*_ increases, see the lower part of Table 2.

#### 5.1.2 Asymmetric toroidal solitons

**Figure 10:**
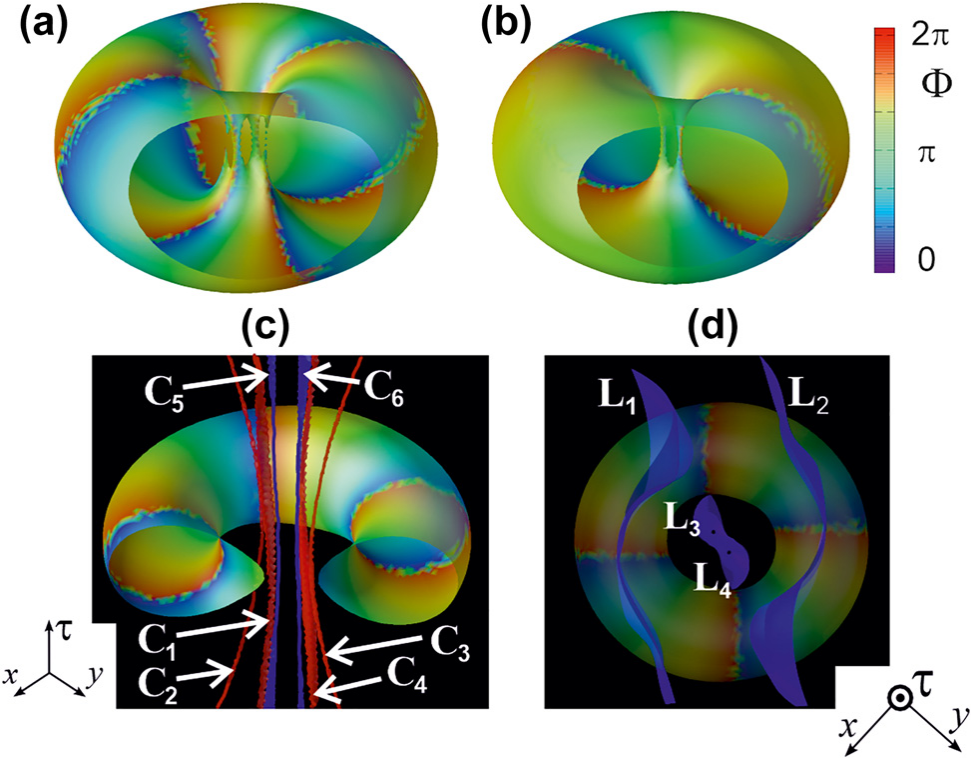
Isointensity surfaces of a toroidal soliton with charges of circular polarization components *m*_+_ = 4, *m*_−_ = −2 (a, b, the front part of the surfaces is not shown) and its polarization singularities: C-lines (c) and L-surfaces (d). There are 4 different vortex lines for the component *E*_+_ $\left(C_{1}-C_{4}\right)$ and 2 for *E*_−_ $\left(C_{5},C_{6}\right)$ . L-surfaces divide the space $x=\left(x,y,\tau \right)$ into 5 regions with different polarization character.

Such somewhat more complex structures arise when the (nonzero) moduli of topological charges of the polarization components differ from each other. We will illustrate them using the example of a also rigid soliton with the initial component charges *m*_+_ = 4, *m*_−_ = −2 (Figure 10). During the establishment process, the initially degenerate vortex lines of the components split into lines with single charges and the axial symmetry of the intensity distribution is lost.

**Figure 11:**
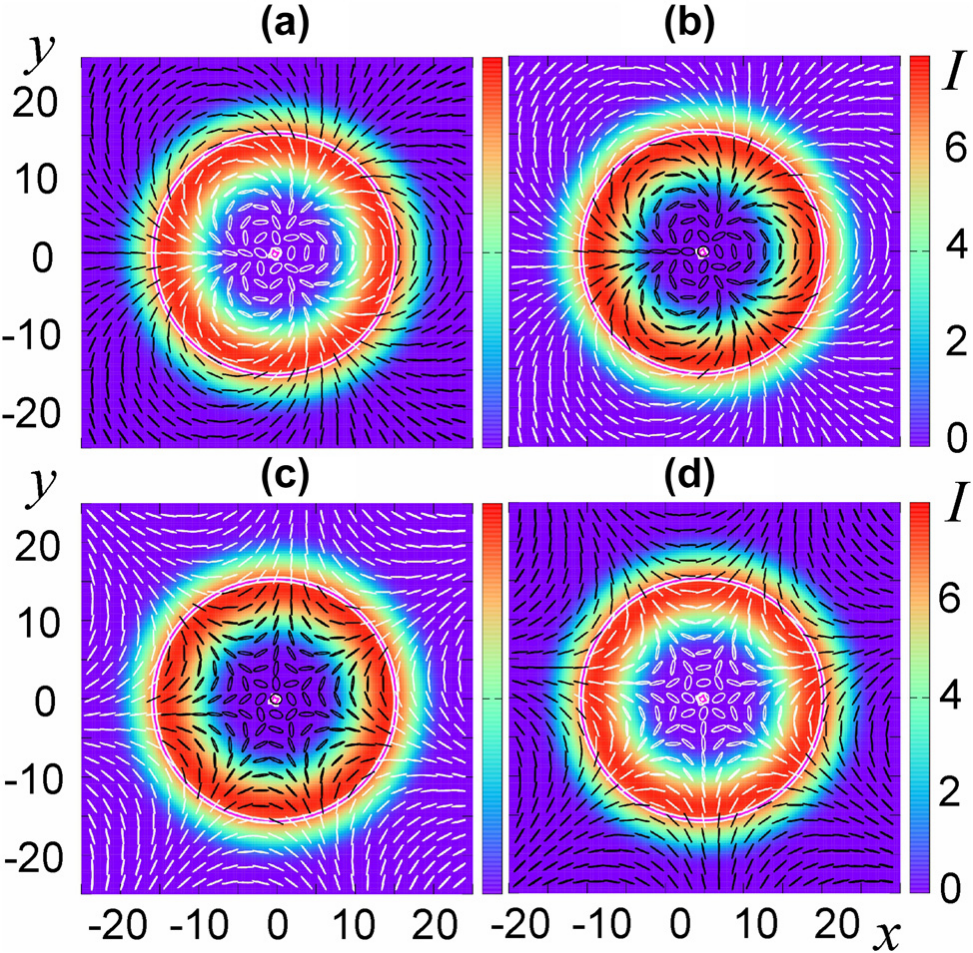
Distributions of total intensity and polarization ellipses of the original symmetric toroidal soliton (a) and its “twins” (b–d). Topological characteristics: *m*_+_ = 3, *m*_−_ = −2, *η* = 5/2 (a), *m*_+_ = 2, *m*_−_ = −3, *η* = 5/2 (b), *m*_+_ = −2, *m*_−_ = 3, *η* = −5/2 (c), *m*_+_ = −3, *m*_−_ = 2, *η* = −5/2 (d).

The existence of a soliton of the type under consideration also implies the existence of its “doubles”, which we will illustrate using the example of a soliton with the initial topological charges of the polarization components *m*_+_ = 3, *m*_−_ = −2 and, accordingly, the Poincaré index *η* = 5/2 (Figure 11). When the signs of both topological charges change, *m*_+_ = −3, *m*_−_ = 2, the sign of the Poincaré index also changes, which is equivalent to the flip of the soliton in space. When the charges of the components are permuted, *m*_+_ = −2, *m*_−_ = 3, the signs of the Poincaré index and the Stokes parameter *s*_3_ change simultaneously. It follows from the latter that the right and left elliptical polarizations change places. When the charges are permuted and their signs simultaneously change, *m*_+_ = 2, *m*_−_ = −3, the Poincaré index retains its sign, but the Stokes parameter *s*_3_ changes it with the consequences indicated above.

The stability regions in the parameter space of these types of solitons were tested both numerically by directly solving the governing equation and approximately semi-analytically. These regions, as a rule, narrow with increasing of topological charges. This issue, as well as destabilization scenarios when leaving the stability region, are discussed in some more detail further in Section 5.2.

### 5.2 Cruciform solitons

**Figure 12:**
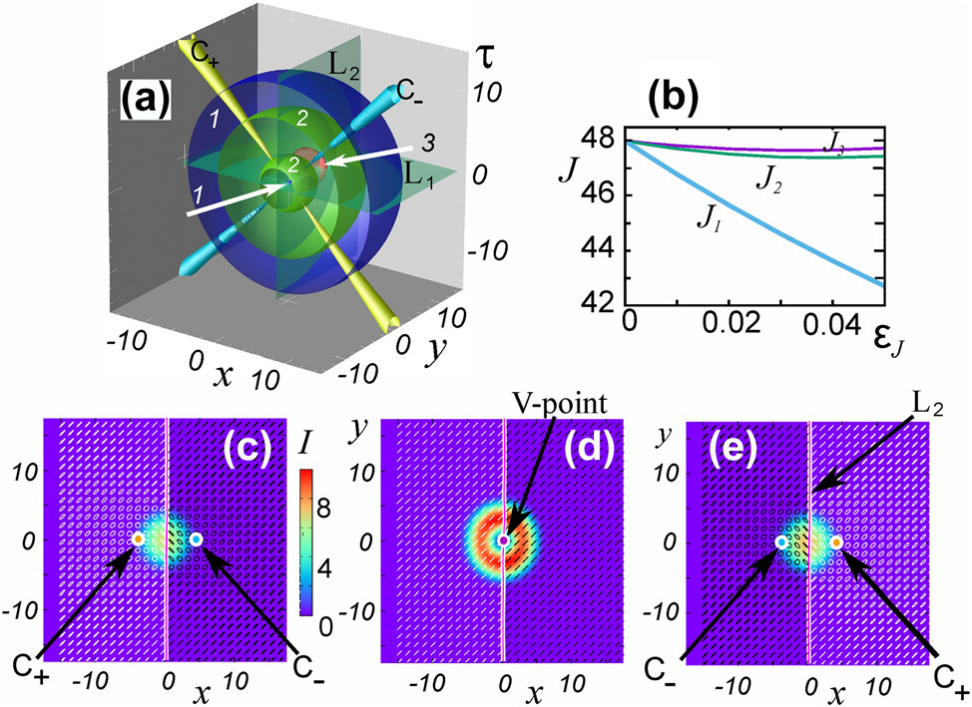
Singularities of cruciform solitons. (a) Surfaces with total intensity *I* = 0.1 (blue, 1), 5 (green, 2) and 8.96 (red, 3); (b) three principle moments of inertia depending from *ɛ*_*J*_; (c-e) polarization structure and total intensity profile at *τ* = 8, 0 and −8.

At supercritical angles of inclination of the symmetry axes of the generating scalar “tori” with charges, another type of solitons is formed, which we will call cruciform. In this case, the soliton is also rigid, but its two vortex lines, also known as C-lines, are approximately orthogonal. The lines intersect at the center of the soliton, which is the V-point, see Figure 12(a). In addition to these lines, there are two L-surfaces, *L*_1_ and *L*_2_, which at small *ɛ*_*J*_ are close to mutually orthogonal planes.

**Figure 13:**
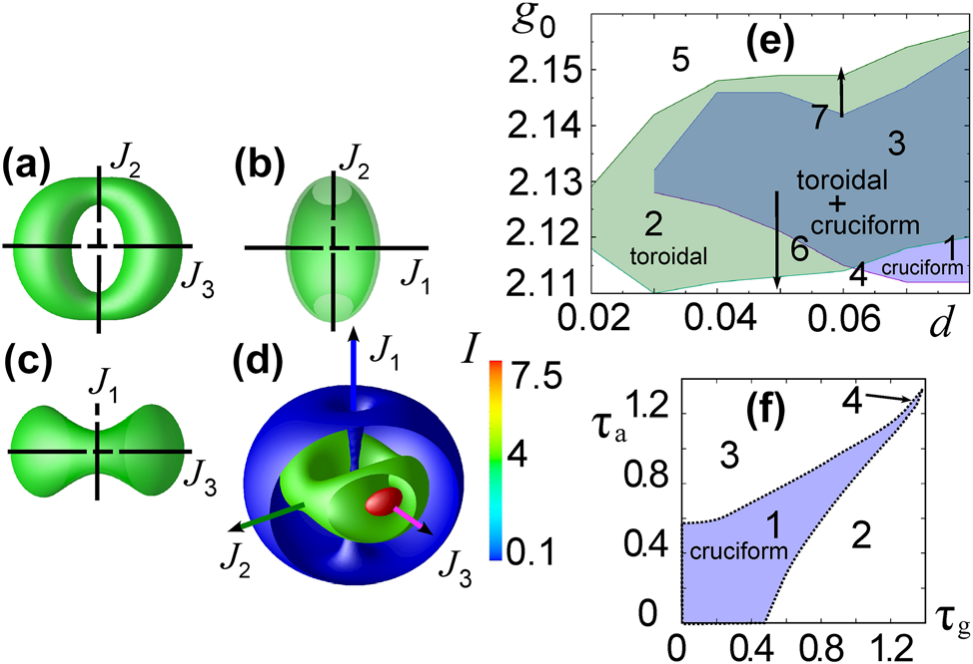
Domains of stability and effect of relaxation for 3D solitons. (a–c) Isointensity surfaces of circular components in three projections. Dash-dotted lines are sections of the symmetry planes. (d) Isointensity surfaces at three levels in isomerism. *J*_1−3_ are the principal values of the tensor of inertia, they also show the principal axes of inertia. (e, f) Domains of stability of toroidal (e) and cruciform (e, f) soliton with topological charges of the circular polarization components *m*_+_ = 1, *m*_−_ = −1 on the planes of parameters *d*, *g*_0_ (e) and *τ*_*g*_, *τ*_*a*_ (f). The vertical dashed lines with arrows in (e) show scenarios of destabilization of a toroidal soliton.

Figure 13(e) shows the stability regions of the cruciform and toroidal solitons with charges *m*_+_ = 1, *m*_−_ = −1 in the plane of coefficients *d* and *g*_0_. These regions intersect significantly, but do not coincide. The same figure explains the scenarios of soliton destabilization when going beyond the boundaries of the stability region.

### 5.3 Additional factors

#### 5.3.1 Relaxation

Relaxation processes, which are essential for class B lasers, are taken into account using the Bloch equation (4) for amplifying media and equation (3) for a saturable absorber. The stability region of a cruciform soliton with charges in the plane of parameters of the relaxation times of the amplifier *τ*_*g*_ and absorber *τ*_*a*_ is shown in Figure 13(f). The figure confirms the stability of the soliton at small relaxation times, when the inertialess approximation for the medium is justified. In addition, stability is also preserved at significant values of relaxation times, provided that *τ*_*a*_ ≈ *τ*_*g*_.

More precisely, in region 1 in this figure there are also weakly modulated solitons. When the parameters leave region 1 and enter 2, the modulation depth increases and then the soliton disappears with the establishment of a non-lasing mode everywhere. Entering region 3 leads either to a transition to spatially uniform generation or to a non-lasing mode. For parameters in the vicinity of 4, a transition to a toroidal soliton occurs.

#### 5.3.2 Anisotropy of diffusion coefficients

**Figure 14:**
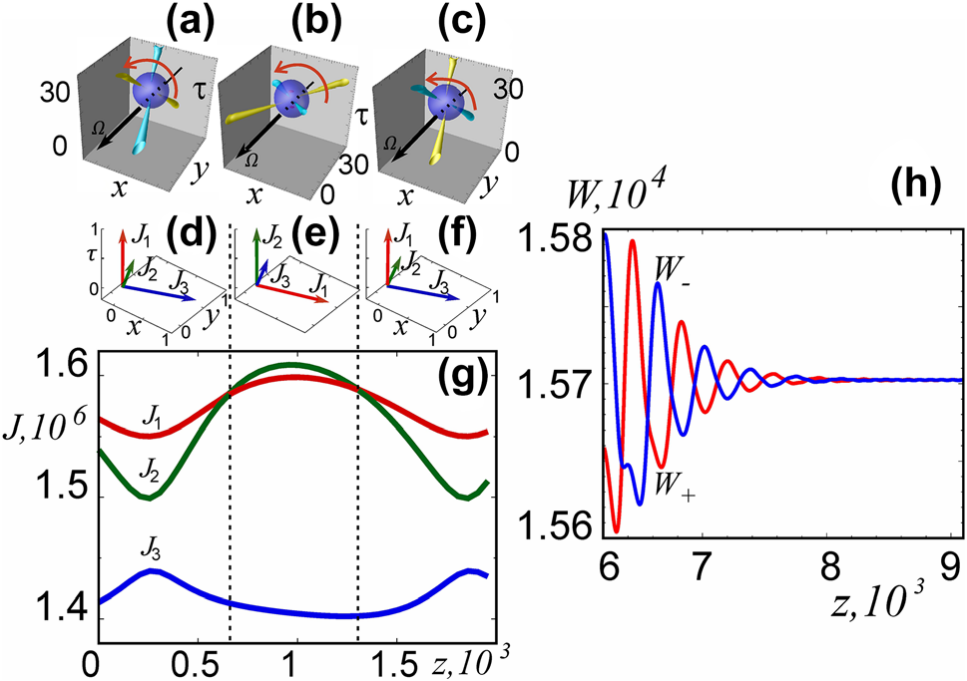
Characteristics of a cruciform soliton when all three diffusion coefficients differ, *d*_*x*_ = 2.138, *d*_*y*_ = 0.01, *d*_*τ*_ = 0.15. (a–f) Three variants of direction of axes of soliton rotations. (g) Variation of the circular components energy during the rotation for these variants. (h) Variation of energies of the circular components during the transient.

Isotropy of space $\left(x,y,\tau \right)$ is violated when taking into account the natural difference between the diffusion coefficients *d*_*τ*_ and *d*_⊥_ = *d*_*x*_ = *d*_*y*_. In this case, the orientation of the soliton in this space is no longer indifferent, since the distinguished axis of symmetry *τ* is preserved. In addition, the rotation of the soliton with an increase in the evolutionary variable leads to a modulation of the soliton characteristics. Nevertheless, with antiphase modulation of the energies of the orthogonal polarization components, the total energy of the field gradually comes to a constant value, Figure 14(h).

If all three diffusion coefficients differ significantly, then stable rotation of the soliton is possible around any of the three coordinate axes, Figure 14(a)–(c). Oscillations of energy and moments of inertia with a period equal to half the period of rotation of the soliton are established, Figure 14(g). In this case, the angle between the vortex lines of the circular polarization components is preserved and is close to 90°.

#### 5.3.3 Anisotropy of losses and refractive index of matrix

Such anisotropy of even initially isotropic medium is caused by mechanical stresses. Semiconductor lasers are characterized by the difference in optical properties of the medium in the directions *x* and *y*. Such anisotropy is taken into account in the control equation (1) by an additional term

(19) $$\frac{\partial E_{\pm }}{\partial z}=\left(i+d\right)\Delta E_{\pm }+\left(R-i\;\Theta \right)E_{\mp }+f_{\pm }\left(I,\delta I\right)E_{\pm }.$$

Here *R* shows the difference in the absorption coefficient, and Θ that for the refractive index.

Numerical analysis for the selected parameters of the scheme shows that for nonzero value of Θ even initially symmetric toroidal solitons with linear polarization lose symmetry and become elliptically polarized. When fixing the value Θ = 0 and increasing *R*, there is a very small threshold value *R*_cr_ ∼ 10^−2^, above which the solitons become linearly polarized, *E*_*y*_ = 0.

**Figure 15:**
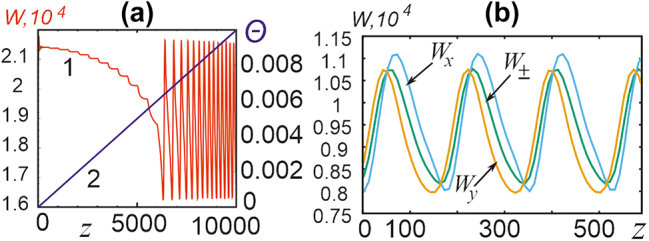
Oscillations of the energy of a toroidal soliton with matrix birefringence. (a) Dependence of the total energy of a soliton on *z* (curve 1, brown) with a linear change in Θ (line 2, blue). (b) Oscillations of the energies of the circular and Cartesian polarization components of the soliton.

Figure 15 illustrates the processes occurring with a toroidal soliton when Θ increases Θ at fixed value *R* = 0. Then, starting with some small threshold value of Θ, the soliton total energy oscillates depending on *z*; the oscillation frequency increases with increasing of Θ, see Figure 15(a). In this case, a periodic change of topological charges of circular polarization components occurs, as Figure 15(b) shows.

Similar oscillations of a cruciform soliton are preceded by a stage of its loss of symmetry. In the end, the cruciform soliton is transformed into an asymmetric toroidal one with oscillations and a periodic change of charges of the circular polarization components.

## 6 Discussion and conclusion

Thus, the inclusion of the polarization degree of freedom significantly expands the family of solitons. Mutual support of the polarization components makes possible the existence and stability of vector solitons under conditions in which their scalar components cannot exist. The increased stability inherent in dissipative solitons makes vector laser solitons promising for a number of applications [27].

First of all, polarization singularities in vector laser solitons can be used to encode information. This is facilitated by the increased stability of laser solitons and the topological security of the information recorded by singularities. At the same time, it is necessary to take into account that both of these arguments are valid only in a certain range of parameters.

While the formation and diagnostics of one-dimensional and two-dimensional radiation structures can be considered technically accessible, three-dimensional structures still require the development of an instrumental base. This, together with a number of fundamental physical problems, hinders the logically natural and necessary advancement toward non-paraxial multidimensional vector laser solitons, including those of extremely short duration, for miniaturization of devices and increase in their productivity [41]. When developing the theory of non-paraxial nonlinear optical structures, it is necessary to abandon the paraxial approximation and limit the analysis to envelope solitons only used in this paper. It seems that such a direction, in demand both in theory and in the applied aspect, will be one of the main ones in the soliton theme. We also point out the insufficiently studied topic of quantum fluctuations of dissipative optical solitons [19], the results of which can characterize the limiting characteristics of these structures.

## References

[1] Kivshar Y. S., Agrawal G. P. (2003). *Optical Solitons: From Fibers to Photonic Crystals*.

[2] Rosanov N. N. (1996). Transverse patterns in wide-aperture nonlinear optical systems. *Prog. Opt.*.

[3] Mandel P. (1997). Theoretical problems in cavity nonlinear optics (hardback). *Cambridge Studies in Modern Optics*.

[4] Rozanov N. N. (2000). Dissipative optical solitons. *Adv. Phys. Sci.*.

[5] Weiss C. O., Trillo S., Torruellas W. (2013). *Spatial Solitons*.

[6] Rosanov N. N. (2002). *Spatial Hysteresis and Optical Patterns*.

[7] Staliunas K., Sanchez-Morcillo V. J. (2003). *Transverse Patterns in Nonlinear Optical Resonators*.

[8] Akhmediev N., Ankiewicz A. (2005). *Dissipative Solitons (Lecture Notes in Physics)*.

[9] Ankiewicz A., Akhmediev N. (2008). *Dissipative Solitons: From Optics to Biology and Medicine*.

[10] Kuszelewicz R., Barbay S., Tissoni G., Almuneau G. (2010). Editorial on dissipative optical solitons. *Eur. Phys. J. D*.

[11] Tlidi M., Staliunas K., Panajotov K., Vladimirov A. G., Clerc M. G. (2014). Localized structures in dissipative media: from optics to plant ecology. *Philos. Trans. R. Soc. A: Math. Phys. Eng. Sci.*.

[12] Lugiato L., Prati F., Brambilla M. (2015). *Nonlinear Optical Systems*.

[13] Grelu P. (2015). *Nonlinear Optical Cavity Dynamics: From Microresonators to Fiber Lasers*.

[14] Tlidi M., Clerc M. G., Panajotov K. (2018). Dissipative structures in matter out of equilibrium: from chemistry, photonics and biology, the legacy of Ilya Prigogine (part 2). *Philos. Trans. R. Soc. A: Math. Phys. Eng. Sci.*.

[15] Kartashov Y. V., Astrakharchik G. E., Malomed B. A., Torner L. (2019). Frontiers in multidimensional self-trapping of nonlinear fields and matter. *Nat. Rev. Phys.*.

[16] Rosanov N. N. (2021). *Dissipative Optical and Related Solitons*.

[17] Malomed B. A. (2022). Multidimensional dissipative solitons and solitary vortices. *Chaos, Solit. Fractals*.

[18] Malomed B. A. (2022). *Multidimensional Solitons*.

[19] Veretenov N. A., Rosanov N. N., Fedorov S. V. (2022). Laser solitons: topological and quantum phenomena. *Phys. Usp.*.

[20] Malomed B. A. (2024). Multidimensional soliton systems. *Adv. Phys. X*.

[21] Mihalache D. (2024). Localized structures in optical media and Bose–Einstein condensates: an overview of recent theoretical and experimental results. *Rom. Rep. Phys.*.

[22] Rozanov N. N., Fedorov S. V. (1992). Diffraction switching waves and autosolitons in a saturable-absorber laser. *Opt Spectrosc.*.

[23] Alfaro-Bittner K., Barbay S., Clerc M. G. (2020). Pulse propagation in a 1D array of excitable semiconductor lasers. *Chaos*.

[24] Soskin M., Boriskina S. V., Chong Y., Dennis M. R., Desyatnikov A. (2016). Singular optics and topological photonics. *J. Opt.*.

[25] Gbur G. J. (2016). *Singular Optics*.

[26] Yin X., Peng C. (2020). Manipulating light radiation from a topological perspective. *Photonics Res.*.

[27] Wang Q., Tu C.-H., Li Y.-N., Wang H.-T. (2021). Polarization singularities: progress, fundamental physics, and prospects. *Appl. Photonics*.

[28] San Miguel M., Feng Q., Moloneym J. V. (1995). Light-polarization dynamics in surface-emitting semiconductor lasers. *Phys. Rev. A*.

[29] Choi H. (2008). Gain recovery dynamics and photon-driven transport in quantum cascade lasers. *Phys. Rev. Lett.*.

[30] Piccardo M. (2019). Frequency-modulated combs obey a variational principle. *Phys. Rev. Lett.*.

[31] Vladimirov A. G., Rozanov N. N., Fedorov S. V., Khodova G. V. (1997). Bifurcation analysis of laser autosolitons. *Quant. Electron.*.

[32] Rosanov N. N., Fedorov S. V., Shatsev A. N. (2005). Two-dimensional laser soliton complexes with weak, strong, and mixed coupling. *Appl. Phys. B*.

[33] Fedorov S. V., Rosanov N. N., Shatsev A. N., Veretenov N. A., Vladimirov A. G. (2003). Topologically multicharged and multihumped rotating solitons in wide-aperture lasers with a saturable absorber. *IEEE J. Quant. Electron.*.

[34] Rosanov N. N., Fedorov S. V., Shatsev A. N., Veretenov N. A., Vladimirov A. G. (2023). Topological semiconductor laser solitons with polarization singularities. *Phys. Rev. A*.

[35] Veretenov N. A., Rosanov N. N., Fedorov S. V. (2023). Topological scalar and vector laser solitons. *Radiophys. Quant. Electron.*.

[36] Panajotov K., Tlidi M. (2018). Localized chaos of elliptically polarized cavity solitons in broad-area VCSEL with a saturable absorber. *Opt. Lett.*.

[37] Veretenov N. A., Fedorov S. V., Rosanov N. N. (2022). Frequency locking and alternation of topological indices of vortex laser solitons. *Opt. Lett.*.

[38] Veretenov N. A., Rosanov N. N., Fedorov S. V. (2023). Toroidal vector dissipative optical solitons with polarization singularities. *Radiophys. Quant. Electron.*.

[39] Veretenov N. A., Rosanov N. N., Fedorov S. V. (2023). Configuration and dynamics of vortex lines of three-dimensional vector laser toroidal solitons. *Opt. Spectrosc.*.

[40] Veretenov N., Fedorov S., Rosanov N. (2024). Dissipative three-dimensional topological optical solitons with crossed localization of polarization components. *Opt. Lett.*.

[41] Rosanov N. N. (2023). Half-cycle electromagnetic pulses and pulse electric area. *Contemp. Phys.*.

